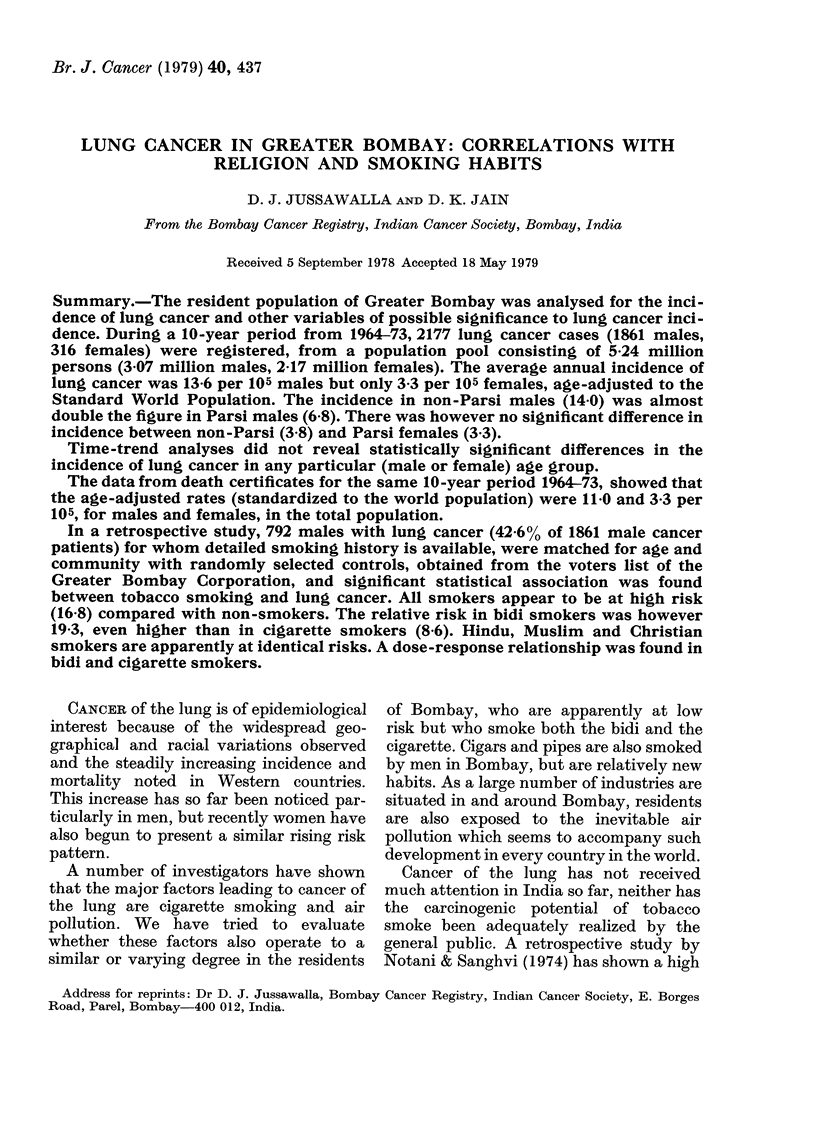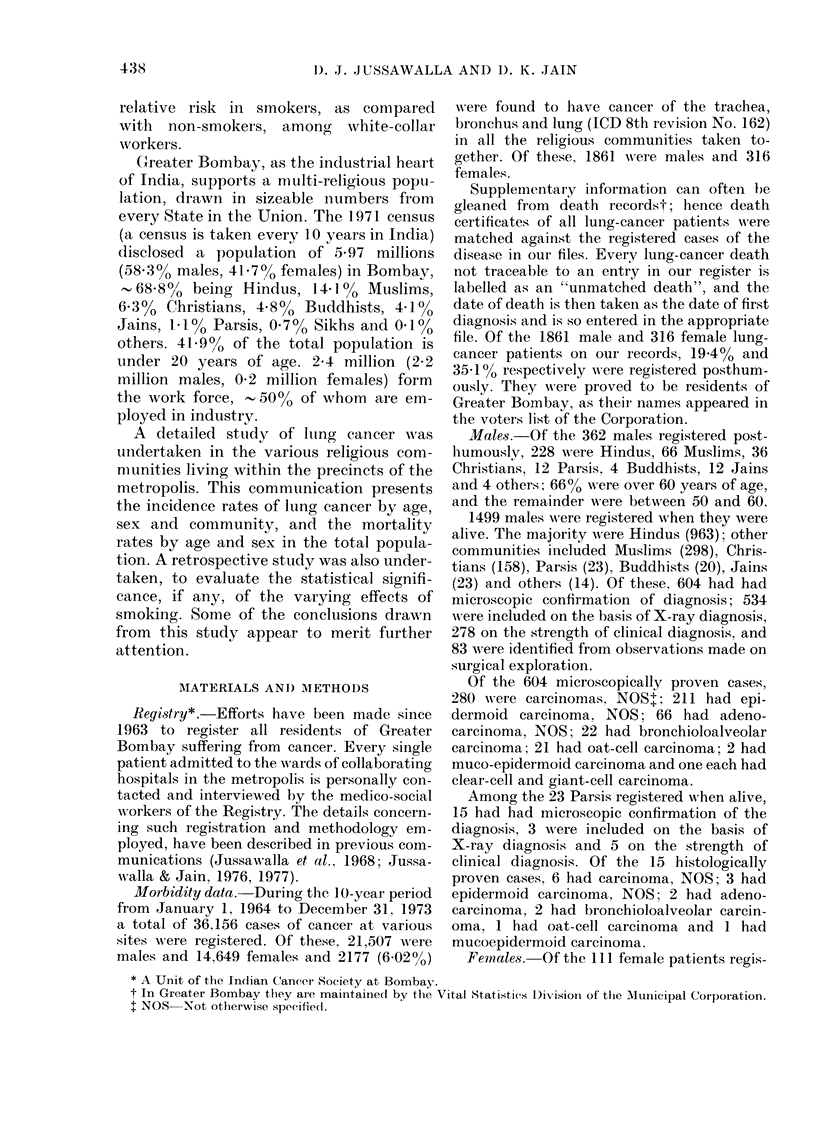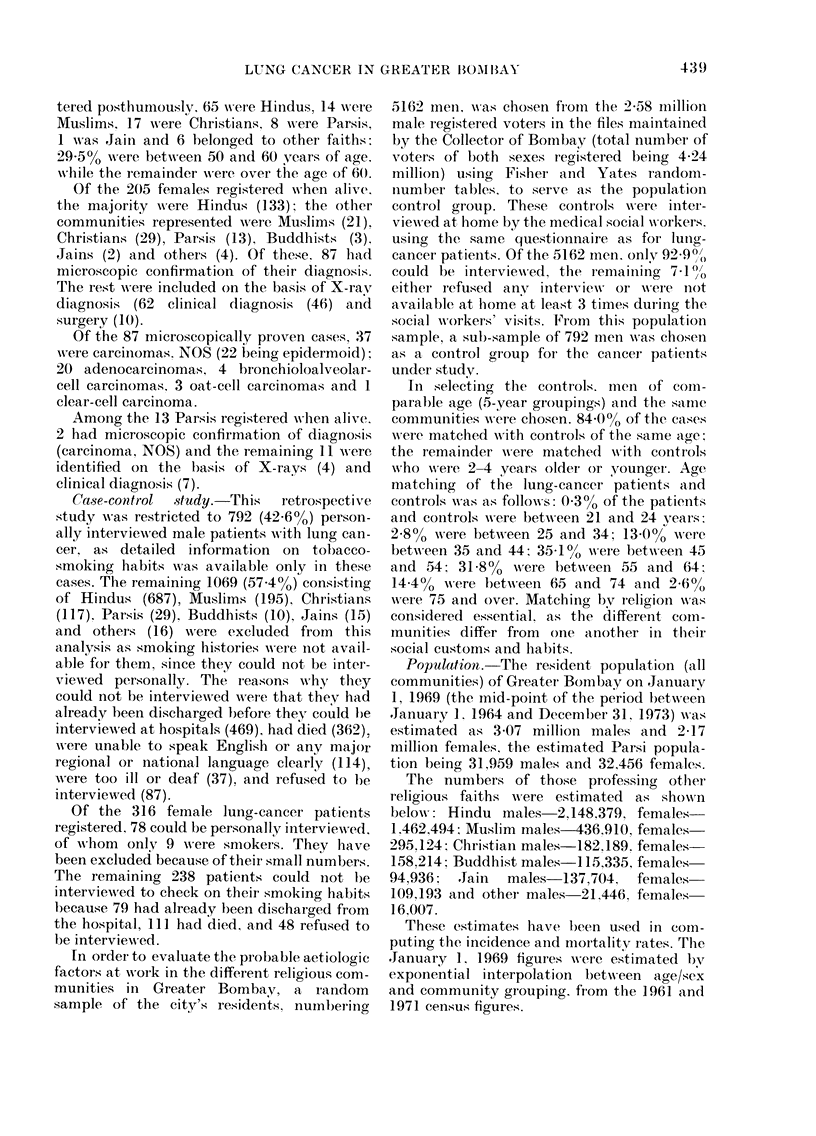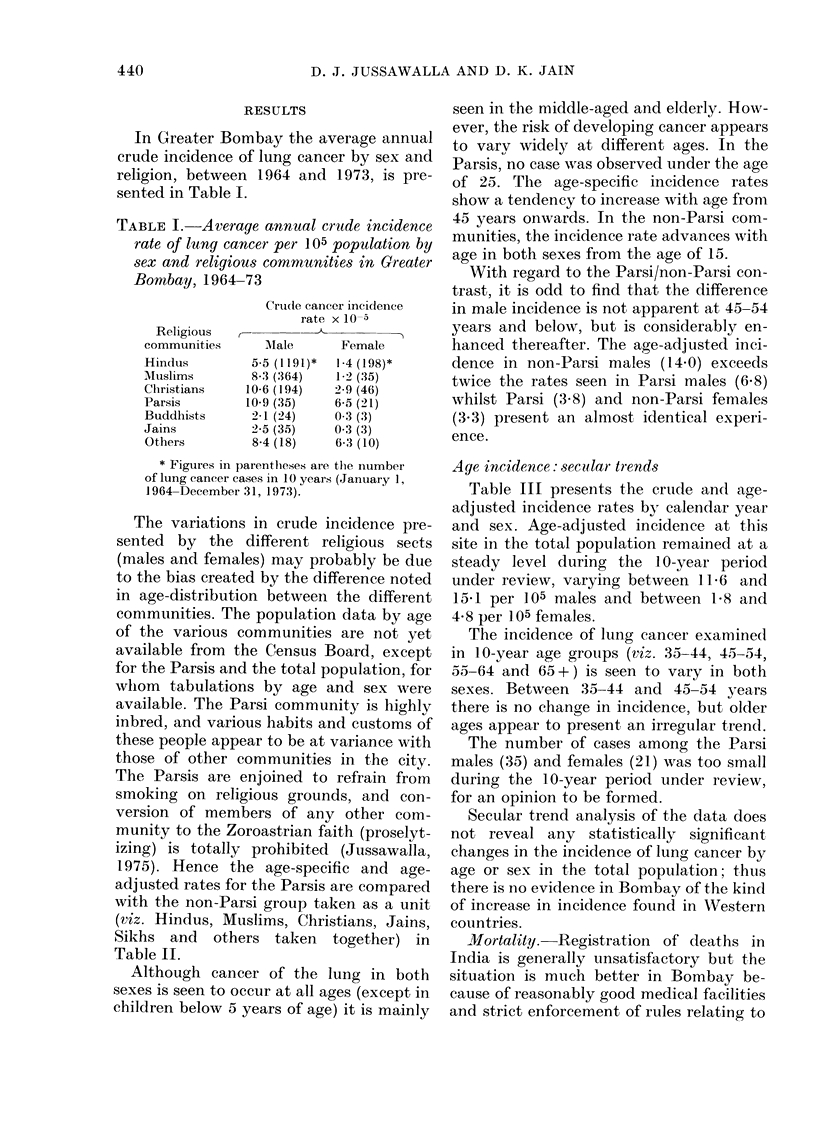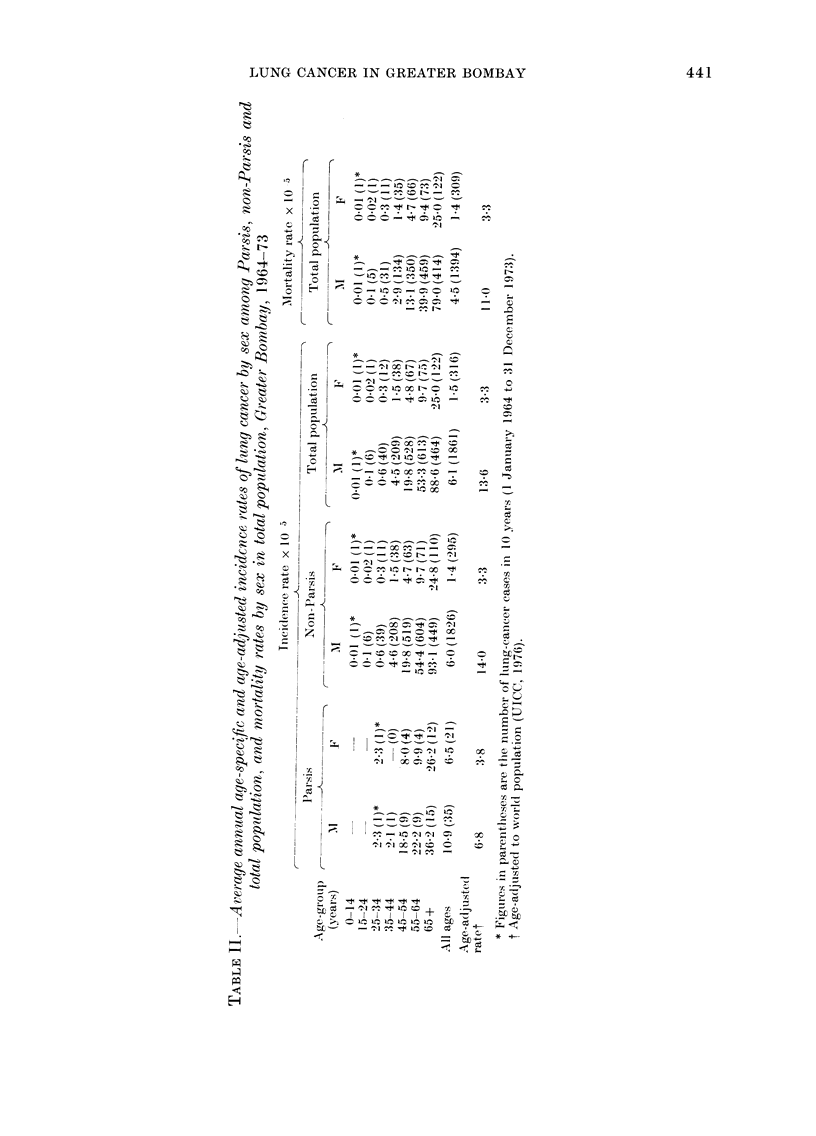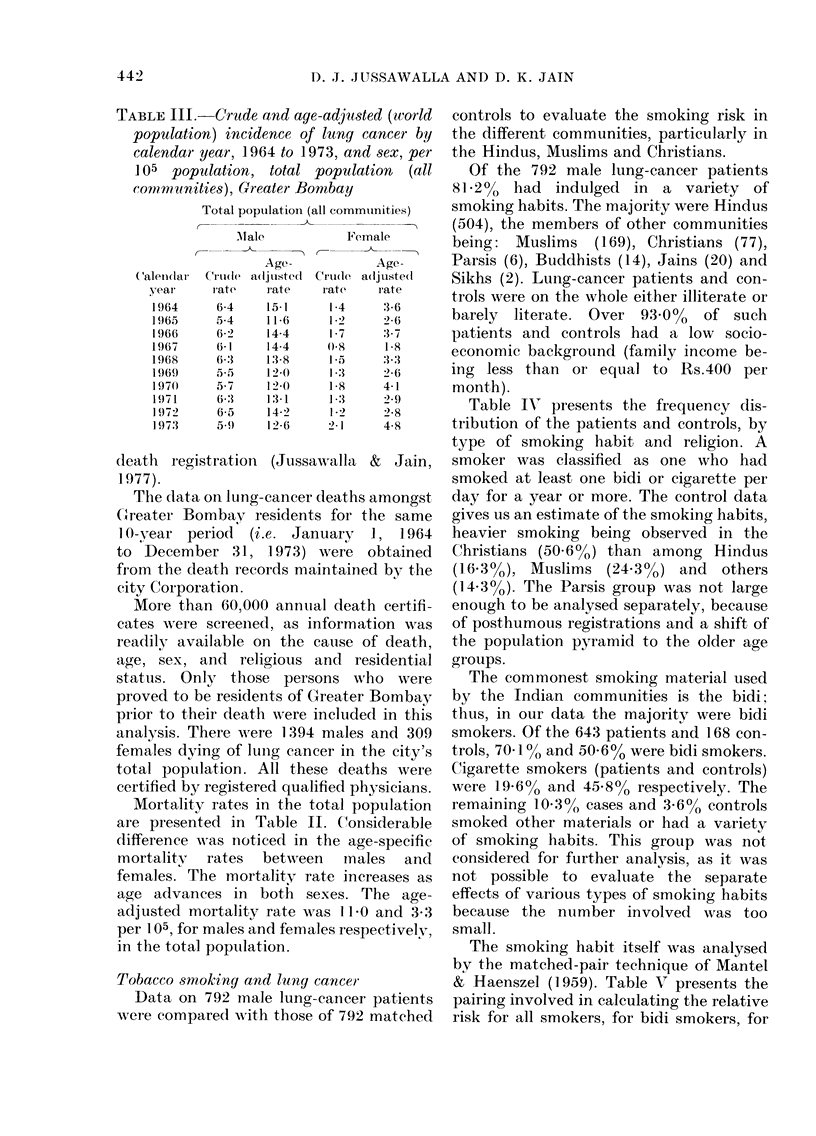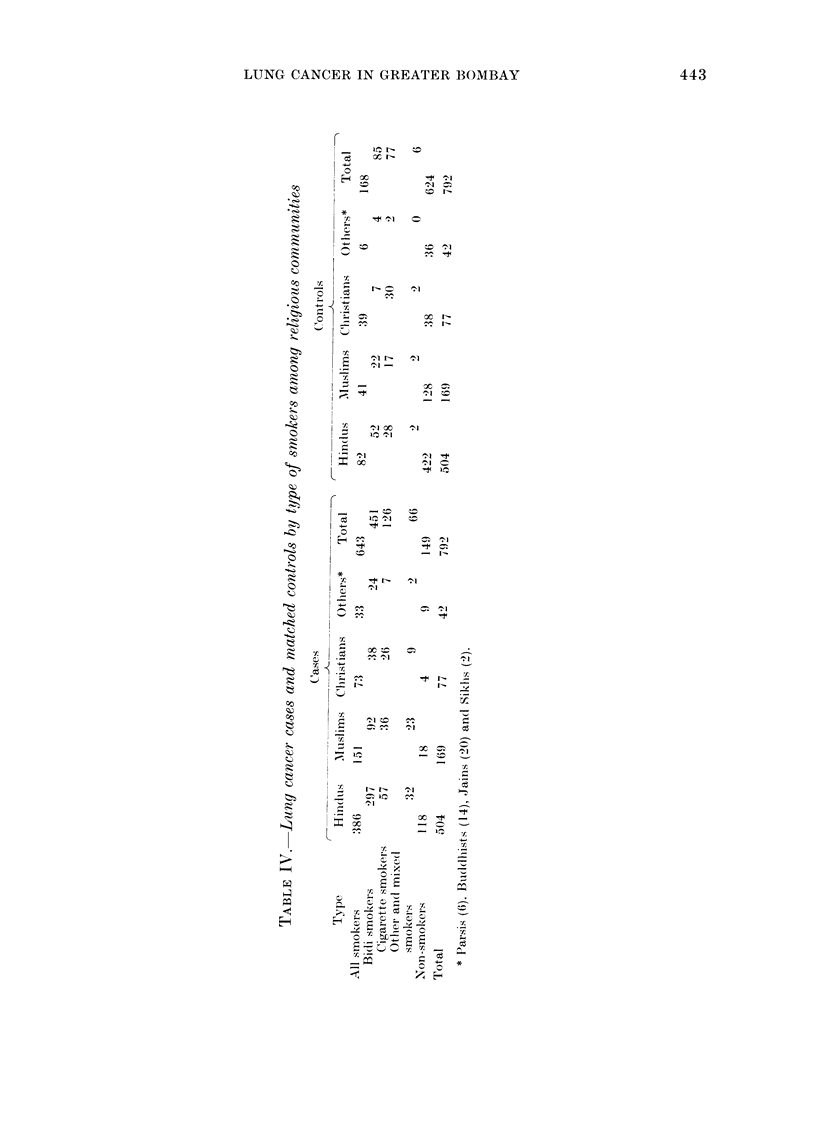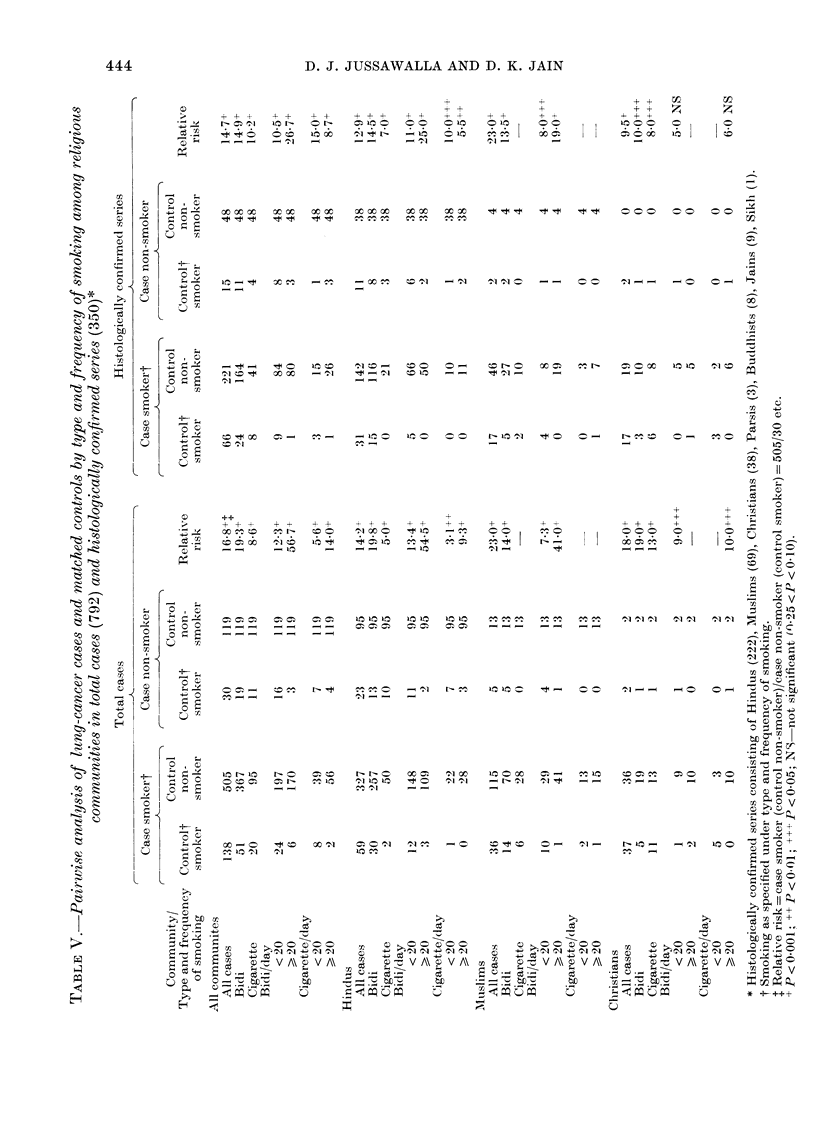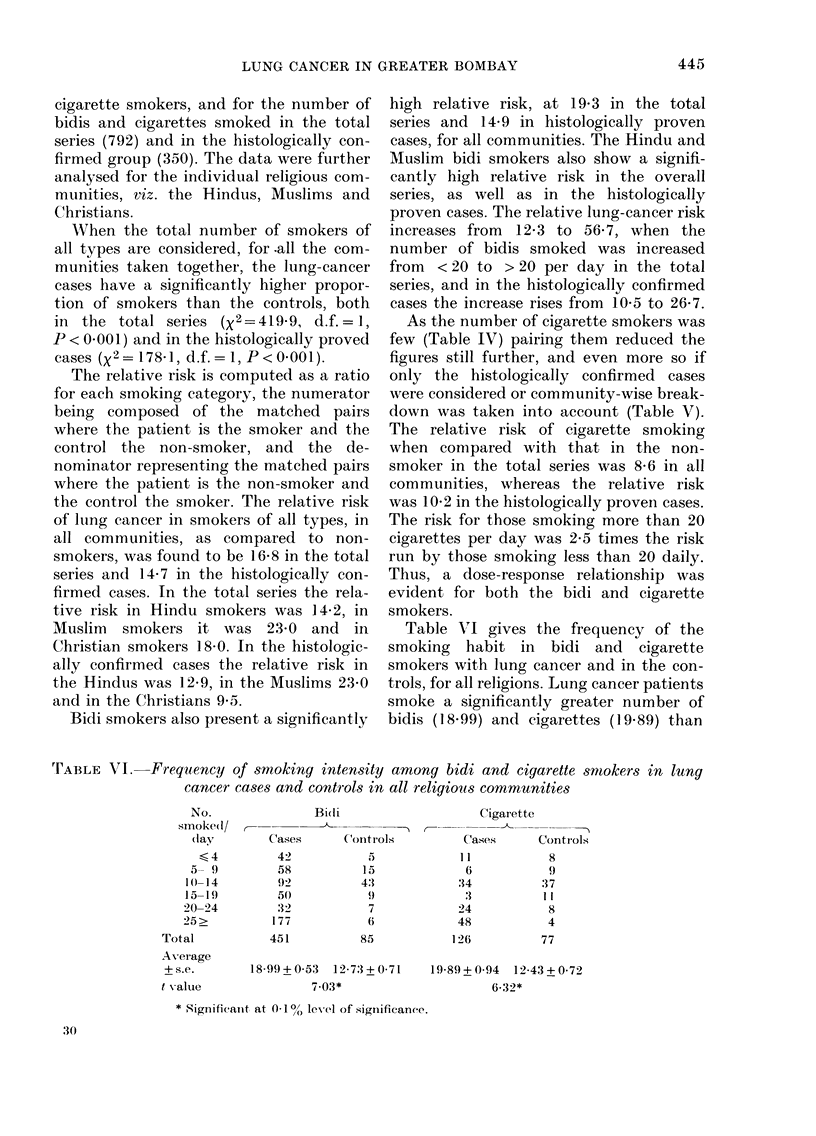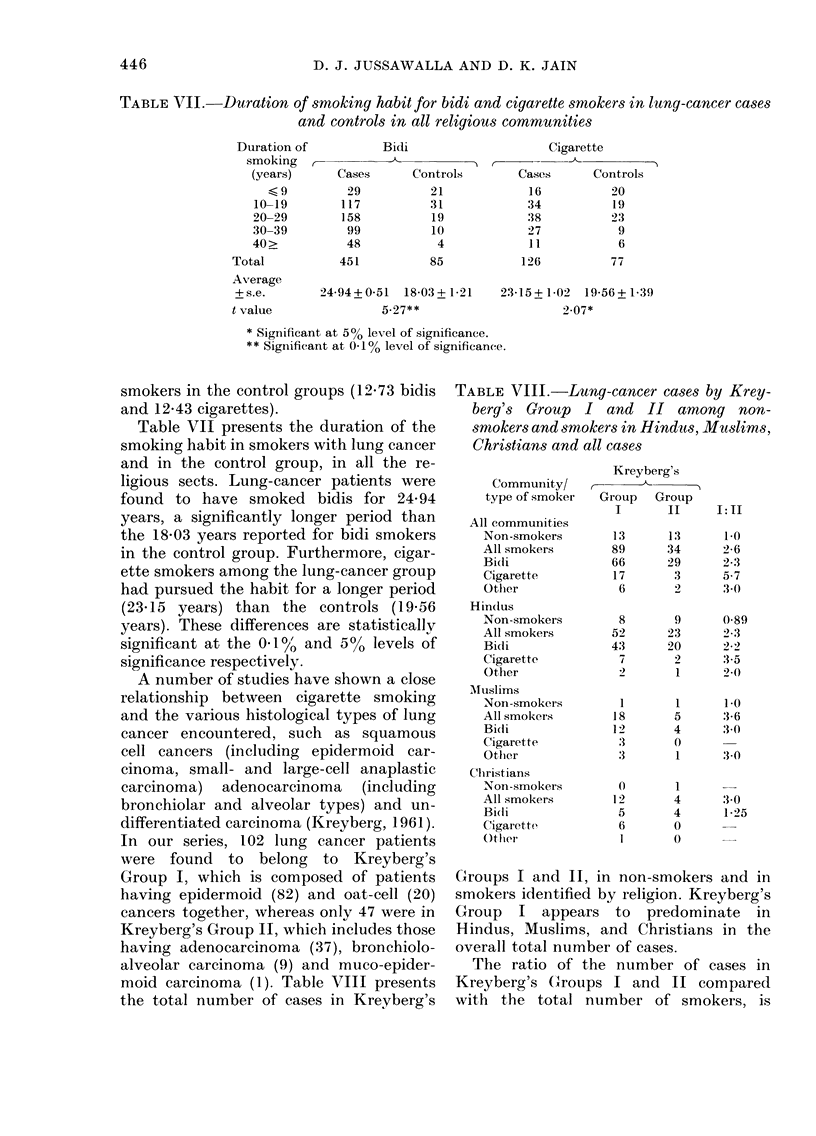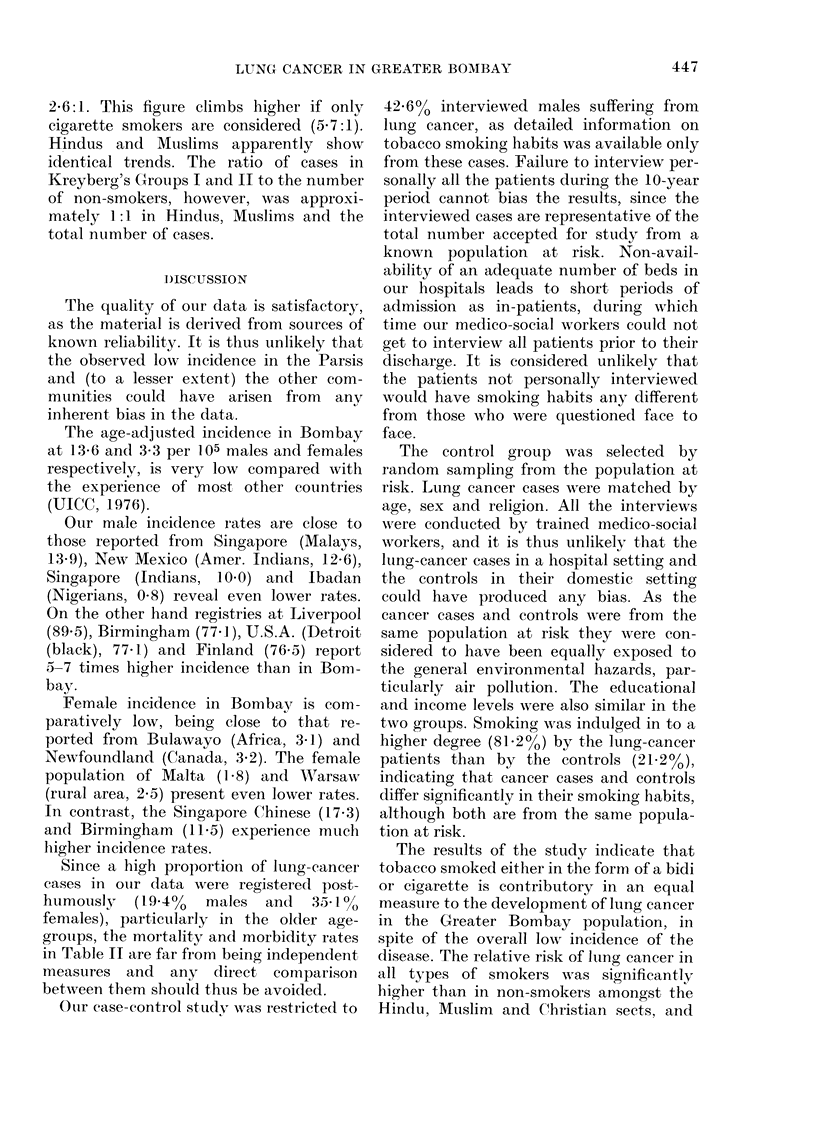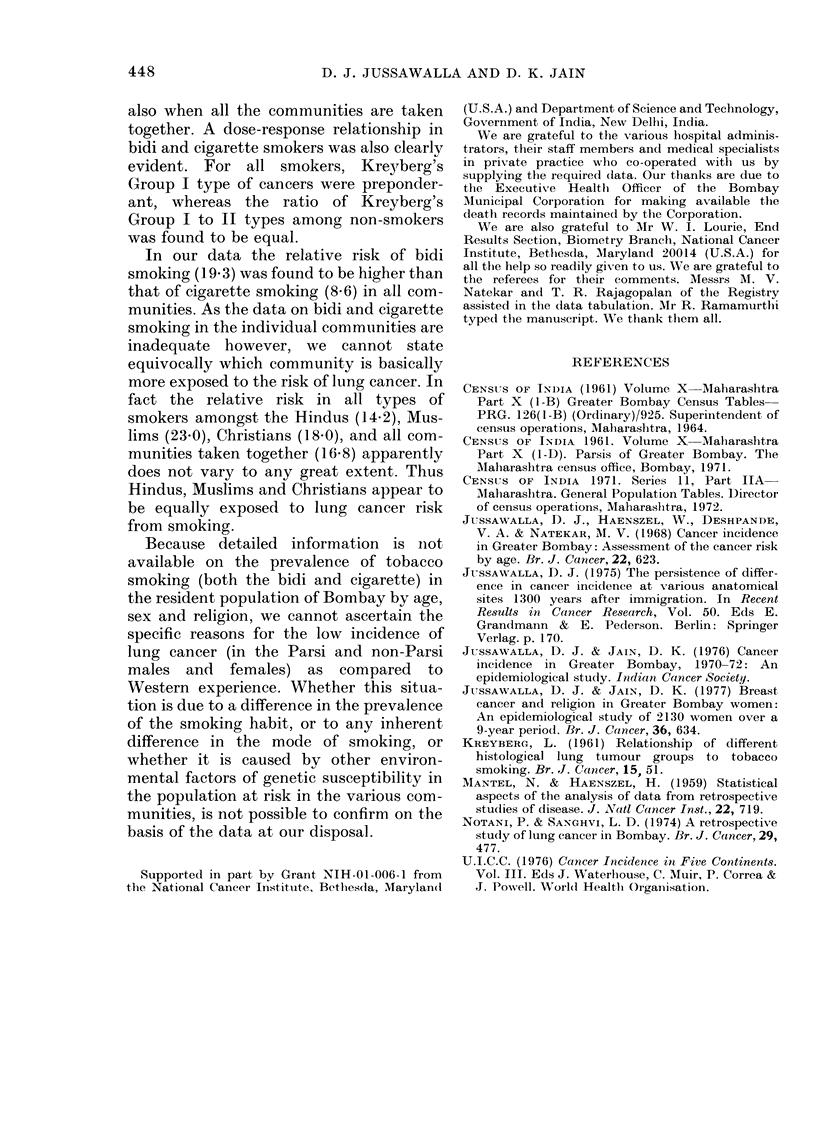# Lung cancer in Greater Bombay: correlations with religion and smoking habits.

**DOI:** 10.1038/bjc.1979.199

**Published:** 1979-09

**Authors:** D. J. Jussawalla, D. K. Jain

## Abstract

The resident population of Greater Bombay was analysed for the incidence of lung cancer and other variables of possible significance to lung cancer incidence. During a 10-year period from 1964-73, 2177 lung cancer cases (1861 males, 316 females) were registered, from a population pool consisting of 5.24 million persons (3.07 million males, 2.17 million females). The average annual incidence of lung cancer was 13.6 per 10(5) males but only 3.3 per 10(5) females, age-adjusted to the Standard World Population. The incidence in non-Parsi males (14.0) was almost double the figure in Parsi males (6.8). There was however no significant difference in incidence between non-Parsi (3.8) and Parsi females (3.3). Time-trend analyses did not reveal statistically significant differences in the incidence of lung cancer in any particular (male or female) age group. The data from death certificates for the same 10-year period 1964-73, showed that the age-adjusted rates (standardized to the world population) were 11.0 and 3.3 per 10(5), for males and females, in the total population. In a retrospective study, 792 males with lung cancer (42.6% of 1861 male cancer patients) for whom detailed smoking history is available, were matched for age and community with randomly selected controls, obtained from the voters list of the Greater Bombay Corporation, and significant statistical association was found between tobacco smoking and lung cancer. All smokers appear to be at high risk (16.8) compared with non-smokers. The relative risk in bidi smokers was however 19.3, even higher than in cigarette smokers (896). Hindu, Muslim and Christian smokers are apparently at identical risks. A dose-reponse relationship was found in bidi and cigarette smokers.


*Br. J. Cancer* (1979) **40**, 437

### LUNG CANCER IN GREATER BOMBAY: CORRELATIONS WITH RELIGION AND SMOKING HABITS

D. J. JUSSAWALLA AND D. K. JAIN

*From the Bombay Cancer Registry, Indian Cancer Society, Bombay, India*

Received 5 September 1978 Accepted 18 May 1979

**Summary.—The resident population of Greater Bombay was analysed for the incidence of lung cancer and other variables of possible significance to lung cancer incidence. During a 10-year period from 1964–73, 2177 lung cancer cases (1861 males, 316 females) were registered, from a population pool consisting of 5·24 million persons (3·07 million males, 2·17 million females). The average annual incidence of lung cancer was 13·6 per $10^5$ males but only 3·3 per $10^5$ females, age-adjusted to the Standard World Population. The incidence in non-Parsi males (14·0) was almost double the figure in Parsi males (6·8). There was however no significant difference in incidence between non-Parsi (3·8) and Parsi females (3·3).**

**Time-trend analyses did not reveal statistically significant differences in the incidence of lung cancer in any particular (male or female) age group.**

**The data from death certificates for the same 10-year period 1964–73, showed that the age-adjusted rates (standardized to the world population) were 11·0 and 3·3 per $10^5$, for males and females, in the total population.**

**In a retrospective study, 792 males with lung cancer (42·6% of 1861 male cancer patients) for whom detailed smoking history is available, were matched for age and community with randomly selected controls, obtained from the voters list of the Greater Bombay Corporation, and significant statistical association was found between tobacco smoking and lung cancer. All smokers appear to be at high risk (16·8) compared with non-smokers. The relative risk in bidi smokers was however 19·3, even higher than in cigarette smokers (8·6). Hindu, Muslim and Christian smokers are apparently at identical risks. A dose-response relationship was found in bidi and cigarette smokers.**

CANCER of the lung is of epidemiological interest because of the widespread geographical and racial variations observed and the steadily increasing incidence and mortality noted in Western countries. This increase has so far been noticed particularly in men, but recently women have also begun to present a similar rising risk pattern.

A number of investigators have shown that the major factors leading to cancer of the lung are cigarette smoking and air pollution. We have tried to evaluate whether these factors also operate to a similar or varying degree in the residents of Bombay, who are apparently at low risk but who smoke both the bidi and the cigarette. Cigars and pipes are also smoked by men in Bombay, but are relatively new habits. As a large number of industries are situated in and around Bombay, residents are also exposed to the inevitable air pollution which seems to accompany such development in every country in the world.

Cancer of the lung has not received much attention in India so far, neither has the carcinogenic potential of tobacco smoke been adequately realized by the general public. A retrospective study by Notani & Sanghvi (1974) has shown a high

Address for reprints: Dr D. J. Jussawalla, Bombay Cancer Registry, Indian Cancer Society, E. Borges Road, Parel, Bombay—400 012, India.

relative risk in smokers, as compared with non-smokers, among white-collar workers.

Greater Bombay, as the industrial heart of India, supports a multi-religious population, drawn in sizeable numbers from every State in the Union. The 1971 census (a census is taken every 10 years in India) disclosed a population of 5·97 millions (58·3% males, 41·7% females) in Bombay, ~68·8% being Hindus, 14·1% Muslims, 6·3% Christians, 4·8% Buddhists, 4·1% Jains, 1·1% Parsis, 0·7% Sikhs and 0·1% others. 41·9% of the total population is under 20 years of age. 2·4 million (2·2 million males, 0·2 million females) form the work force, ~50% of whom are employed in industry.

A detailed study of lung cancer was undertaken in the various religious communities living within the precincts of the metropolis. This communication presents the incidence rates of lung cancer by age, sex and community, and the mortality rates by age and sex in the total population. A retrospective study was also undertaken, to evaluate the statistical significance, if any, of the varying effects of smoking. Some of the conclusions drawn from this study appear to merit further attention.

### MATERIALS AND METHODS

*Registry**.—Efforts have been made since 1963 to register all residents of Greater Bombay suffering from cancer. Every single patient admitted to the wards of collaborating hospitals in the metropolis is personally contacted and interviewed by the medico-social workers of the Registry. The details concerning such registration and methodology employed, have been described in previous communications (Jussawalla *et al.*, 1968; Jussawalla & Jain, 1976, 1977).

*Morbidity data.*—During the 10-year period from January 1, 1964 to December 31, 1973 a total of 36,156 cases of cancer at various sites were registered. Of these, 21,507 were males and 14,649 females and 2177 (6·02%) were found to have cancer of the trachea, bronchus and lung (ICD 8th revision No. 162) in all the religious communities taken together. Of these, 1861 were males and 316 females.

Supplementary information can often be gleaned from death records†; hence death certificates of all lung-cancer patients were matched against the registered cases of the disease in our files. Every lung-cancer death not traceable to an entry in our register is labelled as an "unmatched death", and the date of death is then taken as the date of first diagnosis and is so entered in the appropriate file. Of the 1861 male and 316 female lung-cancer patients on our records, 19·4% and 35·1% respectively were registered posthumously. They were proved to be residents of Greater Bombay, as their names appeared in the voters list of the Corporation.

*Males.*—Of the 362 males registered posthumously, 228 were Hindus, 66 Muslims, 36 Christians, 12 Parsis, 4 Buddhists, 12 Jains and 4 others; 66% were over 60 years of age, and the remainder were between 50 and 60.

1499 males were registered when they were alive. The majority were Hindus (963); other communities included Muslims (298), Christians (158), Parsis (23), Buddhists (20), Jains (23) and others (14). Of these, 604 had had microscopic confirmation of diagnosis; 534 were included on the basis of X-ray diagnosis, 278 on the strength of clinical diagnosis, and 83 were identified from observations made on surgical exploration.

Of the 604 microscopically proven cases, 280 were carcinomas, NOS‡; 211 had epidermoid carcinoma, NOS; 66 had adenocarcinoma, NOS; 22 had bronchioloalveolar carcinoma; 21 had oat-cell carcinoma; 2 had muco-epidermoid carcinoma and one each had clear-cell and giant-cell carcinoma.

Among the 23 Parsis registered when alive, 15 had had microscopic confirmation of the diagnosis, 3 were included on the basis of X-ray diagnosis and 5 on the strength of clinical diagnosis. Of the 15 histologically proven cases, 6 had carcinoma, NOS; 3 had epidermoid carcinoma, NOS; 2 had adenocarcinoma, 2 had bronchioloalveolar carcinoma, 1 had oat-cell carcinoma and 1 had mucoepidermoid carcinoma.

*Females.*—Of the 111 female patients regis-

* A Unit of the Indian Cancer Society at Bombay.
† In Greater Bombay they are maintained by the Vital Statistics Division of the Municipal Corporation.
‡ NOS—Not otherwise specified.

tered posthumously, 65 were Hindus, 14 were Muslims, 17 were Christians, 8 were Parsis, 1 was Jain and 6 belonged to other faiths; 29·5% were between 50 and 60 years of age, while the remainder were over the age of 60.

Of the 205 females registered when alive, the majority were Hindus (133); the other communities represented were Muslims (21), Christians (29), Parsis (13), Buddhists (3), Jains (2) and others (4). Of these, 87 had microscopic confirmation of their diagnosis. The rest were included on the basis of X-ray diagnosis (62 clinical diagnosis (46) and surgery (10).

Of the 87 microscopically proven cases, 37 were carcinomas, NOS (22 being epidermoid); 20 adenocarcinomas, 4 bronchioloalveolar-cell carcinomas, 3 oat-cell carcinomas and 1 clear-cell carcinoma.

Among the 13 Parsis registered when alive, 2 had microscopic confirmation of diagnosis (carcinoma, NOS) and the remaining 11 were identified on the basis of X-rays (4) and clinical diagnosis (7).

*Case-control study.*—This retrospective study was restricted to 792 (42·6%) personally interviewed male patients with lung cancer, as detailed information on tobacco-smoking habits was available only in these cases. The remaining 1069 (57·4%) consisting of Hindus (687), Muslims (195), Christians (117), Parsis (29), Buddhists (10), Jains (15) and others (16) were excluded from this analysis as smoking histories were not available for them, since they could not be interviewed personally. The reasons why they could not be interviewed were that they had already been discharged before they could be interviewed at hospitals (469), had died (362), were unable to speak English or any major regional or national language clearly (114), were too ill or deaf (37), and refused to be interviewed (87).

Of the 316 female lung-cancer patients registered, 78 could be personally interviewed, of whom only 9 were smokers. They have been excluded because of their small numbers. The remaining 238 patients could not be interviewed to check on their smoking habits because 79 had already been discharged from the hospital, 111 had died, and 48 refused to be interviewed.

In order to evaluate the probable aetiologic factors at work in the different religious communities in Greater Bombay, a random sample of the city's residents, numbering 5162 men, was chosen from the 2·58 million male registered voters in the files maintained by the Collector of Bombay (total number of voters of both sexes registered being 4·24 million) using Fisher and Yates random-number tables, to serve as the population control group. These controls were interviewed at home by the medical social workers, using the same questionnaire as for lung-cancer patients. Of the 5162 men, only 92·9% could be interviewed, the remaining 7·1% either refused any interview or were not available at home at least 3 times during the social workers' visits. From this population sample, a sub-sample of 792 men was chosen as a control group for the cancer patients under study.

In selecting the controls, men of comparable age (5-year groupings) and the same communities were chosen. 84·0% of the cases were matched with controls of the same age; the remainder were matched with controls who were 2–4 years older or younger. Age matching of the lung-cancer patients and controls was as follows: 0·3% of the patients and controls were between 21 and 24 years; 2·8% were between 25 and 34; 13·0% were between 35 and 44; 35·1% were between 45 and 54; 31·8% were between 55 and 64; 14·4% were between 65 and 74 and 2·6% were 75 and over. Matching by religion was considered essential, as the different communities differ from one another in their social customs and habits.

*Population.*—The resident population (all communities) of Greater Bombay on January 1, 1969 (the mid-point of the period between January 1, 1964 and December 31, 1973) was estimated as 3·07 million males and 2·17 million females, the estimated Parsi population being 31,959 males and 32,456 females.

The numbers of those professing other religious faiths were estimated as shown below: Hindu males—2,148,379, females—1,462,494; Muslim males—436,910, females—295,124; Christian males—182,189, females—158,214; Buddhist males—115,335, females—94,936; Jain males—137,704, females—109,193 and other males—21,446, females—16,007.

These estimates have been used in computing the incidence and mortality rates. The January 1, 1969 figures were estimated by exponential interpolation between age/sex and community grouping, from the 1961 and 1971 census figures.

### RESULTS

In Greater Bombay the average annual crude incidence of lung cancer by sex and religion, between 1964 and 1973, is presented in Table I.

TABLE I.—*Average annual crude incidence rate of lung cancer per* $10^5$ *population by sex and religious communities in Greater Bombay*, 1964–73

| Religious communities | Crude cancer incidence rate $\times 10^{-5}$ | |
|---|---|---|
| | Male | Female |
| Hindus | 5·5 (1191)* | 1·4 (198)* |
| Muslims | 8·3 (364) | 1·2 (35) |
| Christians | 10·6 (194) | 2·9 (46) |
| Parsis | 10·9 (35) | 6·5 (21) |
| Buddhists | 2·1 (24) | 0·3 (3) |
| Jains | 2·5 (35) | 0·3 (3) |
| Others | 8·4 (18) | 6·3 (10) |

* Figures in parentheses are the number of lung cancer cases in 10 years (January 1, 1964–December 31, 1973).

The variations in crude incidence presented by the different religious sects (males and females) may probably be due to the bias created by the difference noted in age-distribution between the different communities. The population data by age of the various communities are not yet available from the Census Board, except for the Parsis and the total population, for whom tabulations by age and sex were available. The Parsi community is highly inbred, and various habits and customs of these people appear to be at variance with those of other communities in the city. The Parsis are enjoined to refrain from smoking on religious grounds, and conversion of members of any other community to the Zoroastrian faith (proselytizing) is totally prohibited (Jussawalla, 1975). Hence the age-specific and age-adjusted rates for the Parsis are compared with the non-Parsi group taken as a unit (*viz.* Hindus, Muslims, Christians, Jains, Sikhs and others taken together) in Table II.

Although cancer of the lung in both sexes is seen to occur at all ages (except in children below 5 years of age) it is mainly seen in the middle-aged and elderly. However, the risk of developing cancer appears to vary widely at different ages. In the Parsis, no case was observed under the age of 25. The age-specific incidence rates show a tendency to increase with age from 45 years onwards. In the non-Parsi communities, the incidence rate advances with age in both sexes from the age of 15.

With regard to the Parsi/non-Parsi contrast, it is odd to find that the difference in male incidence is not apparent at 45–54 years and below, but is considerably enhanced thereafter. The age-adjusted incidence in non-Parsi males (14·0) exceeds twice the rates seen in Parsi males (6·8) whilst Parsi (3·8) and non-Parsi females (3·3) present an almost identical experience.

### *Age incidence: secular trends*

Table III presents the crude and age-adjusted incidence rates by calendar year and sex. Age-adjusted incidence at this site in the total population remained at a steady level during the 10-year period under review, varying between 11·6 and 15·1 per $10^5$ males and between 1·8 and 4·8 per $10^5$ females.

The incidence of lung cancer examined in 10-year age groups (*viz.* 35–44, 45–54, 55–64 and 65+) is seen to vary in both sexes. Between 35–44 and 45–54 years there is no change in incidence, but older ages appear to present an irregular trend.

The number of cases among the Parsi males (35) and females (21) was too small during the 10-year period under review, for an opinion to be formed.

Secular trend analysis of the data does not reveal any statistically significant changes in the incidence of lung cancer by age or sex in the total population; thus there is no evidence in Bombay of the kind of increase in incidence found in Western countries.

*Mortality.*—Registration of deaths in India is generally unsatisfactory but the situation is much better in Bombay because of reasonably good medical facilities and strict enforcement of rules relating to

TABLE III.—*Crude and age-adjusted (world population) incidence of lung cancer by calendar year, 1964 to 1973, and sex, per* $10^5$ *population, total population (all communities), Greater Bombay*

| | Total population (all communities) | | | |
|---|---|---|---|---|
| | Male | | Female | |
| Calendar year | Crude rate | Age-adjusted rate | Crude rate | Age-adjusted rate |
| 1964 | 6·4 | 15·1 | 1·4 | 3·6 |
| 1965 | 5·4 | 11·6 | 1·2 | 2·6 |
| 1966 | 6·2 | 14·4 | 1·7 | 3·7 |
| 1967 | 6·1 | 14·4 | 0·8 | 1·8 |
| 1968 | 6·3 | 13·8 | 1·5 | 3·3 |
| 1969 | 5·5 | 12·0 | 1·3 | 2·6 |
| 1970 | 5·7 | 12·0 | 1·8 | 4·1 |
| 1971 | 6·3 | 13·1 | 1·3 | 2·9 |
| 1972 | 6·5 | 14·2 | 1·2 | 2·8 |
| 1973 | 5·9 | 12·6 | 2·1 | 4·8 |

death registration (Jussawalla & Jain, 1977).

The data on lung-cancer deaths amongst Greater Bombay residents for the same 10-year period (*i.e.* January 1, 1964 to December 31, 1973) were obtained from the death records maintained by the city Corporation.

More than 60,000 annual death certificates were screened, as information was readily available on the cause of death, age, sex, and religious and residential status. Only those persons who were proved to be residents of Greater Bombay prior to their death were included in this analysis. There were 1394 males and 309 females dying of lung cancer in the city's total population. All these deaths were certified by registered qualified physicians.

Mortality rates in the total population are presented in Table II. Considerable difference was noticed in the age-specific mortality rates between males and females. The mortality rate increases as age advances in both sexes. The age-adjusted mortality rate was 11·0 and 3·3 per $10^5$, for males and females respectively, in the total population.

*Tobacco smoking and lung cancer*

Data on 792 male lung-cancer patients were compared with those of 792 matched controls to evaluate the smoking risk in the different communities, particularly in the Hindus, Muslims and Christians.

Of the 792 male lung-cancer patients 81·2% had indulged in a variety of smoking habits. The majority were Hindus (504), the members of other communities being: Muslims (169), Christians (77), Parsis (6), Buddhists (14), Jains (20) and Sikhs (2). Lung-cancer patients and controls were on the whole either illiterate or barely literate. Over 93·0% of such patients and controls had a low socio-economic background (family income being less than or equal to Rs.400 per month).

Table IV presents the frequency distribution of the patients and controls, by type of smoking habit and religion. A smoker was classified as one who had smoked at least one bidi or cigarette per day for a year or more. The control data gives us an estimate of the smoking habits, heavier smoking being observed in the Christians (50·6%) than among Hindus (16·3%), Muslims (24·3%) and others (14·3%). The Parsis group was not large enough to be analysed separately, because of posthumous registrations and a shift of the population pyramid to the older age groups.

The commonest smoking material used by the Indian communities is the bidi; thus, in our data the majority were bidi smokers. Of the 643 patients and 168 controls, 70·1% and 50·6% were bidi smokers. Cigarette smokers (patients and controls) were 19·6% and 45·8% respectively. The remaining 10·3% cases and 3·6% controls smoked other materials or had a variety of smoking habits. This group was not considered for further analysis, as it was not possible to evaluate the separate effects of various types of smoking habits because the number involved was too small.

The smoking habit itself was analysed by the matched-pair technique of Mantel & Haenszel (1959). Table V presents the pairing involved in calculating the relative risk for all smokers, for bidi smokers, for

* Parsis (6), Buddhists (14), Jains (20) and Sikhs (2).

cigarette smokers, and for the number of bidis and cigarettes smoked in the total series (792) and in the histologically confirmed group (350). The data were further analysed for the individual religious communities, *viz.* the Hindus, Muslims and Christians.

When the total number of smokers of all types are considered, for all the communities taken together, the lung-cancer cases have a significantly higher proportion of smokers than the controls, both in the total series ($\chi^2 = 419 \cdot 9$, d.f. = 1, $P < 0 \cdot 001$) and in the histologically proved cases ($\chi^2 = 178 \cdot 1$, d.f. = 1, $P < 0 \cdot 001$).

The relative risk is computed as a ratio for each smoking category, the numerator being composed of the matched pairs where the patient is the smoker and the control the non-smoker, and the denominator representing the matched pairs where the patient is the non-smoker and the control the smoker. The relative risk of lung cancer in smokers of all types, in all communities, as compared to non-smokers, was found to be 16·8 in the total series and 14·7 in the histologically confirmed cases. In the total series the relative risk in Hindu smokers was 14·2, in Muslim smokers it was 23·0 and in Christian smokers 18·0. In the histologically confirmed cases the relative risk in the Hindus was 12·9, in the Muslims 23·0 and in the Christians 9·5.

Bidi smokers also present a significantly high relative risk, at 19·3 in the total series and 14·9 in histologically proven cases, for all communities. The Hindu and Muslim bidi smokers also show a significantly high relative risk in the overall series, as well as in the histologically proven cases. The relative lung-cancer risk increases from 12·3 to 56·7, when the number of bidis smoked was increased from < 20 to > 20 per day in the total series, and in the histologically confirmed cases the increase rises from 10·5 to 26·7.

As the number of cigarette smokers was few (Table IV) pairing them reduced the figures still further, and even more so if only the histologically confirmed cases were considered or community-wise breakdown was taken into account (Table V). The relative risk of cigarette smoking when compared with that in the non-smoker in the total series was 8·6 in all communities, whereas the relative risk was 10·2 in the histologically proven cases. The risk for those smoking more than 20 cigarettes per day was 2·5 times the risk run by those smoking less than 20 daily. Thus, a dose-response relationship was evident for both the bidi and cigarette smokers.

Table VI gives the frequency of the smoking habit in bidi and cigarette smokers with lung cancer and in the controls, for all religions. Lung cancer patients smoke a significantly greater number of bidis (18·99) and cigarettes (19·89) than

TABLE VI.—*Frequency of smoking intensity among bidi and cigarette smokers in lung cancer cases and controls in all religious communities*

| No. smoked/day | Bidi | | Cigarette | |
|---|---|---|---|---|
| | Cases | Controls | Cases | Controls |
| ≤4 | 42 | 5 | 11 | 8 |
| 5–9 | 58 | 15 | 6 | 9 |
| 10–14 | 92 | 43 | 34 | 37 |
| 15–19 | 50 | 9 | 3 | 11 |
| 20–24 | 32 | 7 | 24 | 8 |
| 25≥ | 177 | 6 | 48 | 4 |
| Total | 451 | 85 | 126 | 77 |
| Average ± s.e. | 18·99 ± 0·53 | 12·73 ± 0·71 | 19·89 ± 0·94 | 12·43 ± 0·72 |
| *t* value | 7·03* | | 6·32* | |

* Significant at 0·1% level of significance.

TABLE VII.—*Duration of smoking habit for bidi and cigarette smokers in lung-cancer cases and controls in all religious communities*

| Duration of smoking (years) | Bidi | | Cigarette | |
|---|---|---|---|---|
| | Cases | Controls | Cases | Controls |
| $\leq 9$ | 29 | 21 | 16 | 20 |
| 10–19 | 117 | 31 | 34 | 19 |
| 20–29 | 158 | 19 | 38 | 23 |
| 30–39 | 99 | 10 | 27 | 9 |
| $40 \geq$ | 48 | 4 | 11 | 6 |
| Total | 451 | 85 | 126 | 77 |
| Average $\pm$ s.e. | $24.94 \pm 0.51$ | $18.03 \pm 1.21$ | $23.15 \pm 1.02$ | $19.56 \pm 1.39$ |
| $t$ value | 5·27** | | 2·07* | |

* Significant at 5% level of significance.
** Significant at 0·1% level of significance.

smokers in the control groups (12·73 bidis and 12·43 cigarettes).

Table VII presents the duration of the smoking habit in smokers with lung cancer and in the control group, in all the religious sects. Lung-cancer patients were found to have smoked bidis for 24·94 years, a significantly longer period than the 18·03 years reported for bidi smokers in the control group. Furthermore, cigarette smokers among the lung-cancer group had pursued the habit for a longer period (23·15 years) than the controls (19·56 years). These differences are statistically significant at the 0·1% and 5% levels of significance respectively.

A number of studies have shown a close relationship between cigarette smoking and the various histological types of lung cancer encountered, such as squamous cell cancers (including epidermoid carcinoma, small- and large-cell anaplastic carcinoma) adenocarcinoma (including bronchiolar and alveolar types) and undifferentiated carcinoma (Kreyberg, 1961). In our series, 102 lung cancer patients were found to belong to Kreyberg's Group I, which is composed of patients having epidermoid (82) and oat-cell (20) cancers together, whereas only 47 were in Kreyberg's Group II, which includes those having adenocarcinoma (37), bronchiolo-alveolar carcinoma (9) and muco-epidermoid carcinoma (1). Table VIII presents the total number of cases in Kreyberg's

TABLE VIII.—*Lung-cancer cases by Kreyberg's Group I and II among non-smokers and smokers in Hindus, Muslims, Christians and all cases*

| Community/ type of smoker | Kreyberg's Group I | Kreyberg's Group II | I:II |
|---|---|---|---|
| All communities | | | |
| Non-smokers | 13 | 13 | 1·0 |
| All smokers | 89 | 34 | 2·6 |
| Bidi | 66 | 29 | 2·3 |
| Cigarette | 17 | 3 | 5·7 |
| Other | 6 | 2 | 3·0 |
| Hindus | | | |
| Non-smokers | 8 | 9 | 0·89 |
| All smokers | 52 | 23 | 2·3 |
| Bidi | 43 | 20 | 2·2 |
| Cigarette | 7 | 2 | 3·5 |
| Other | 2 | 1 | 2·0 |
| Muslims | | | |
| Non-smokers | 1 | 1 | 1·0 |
| All smokers | 18 | 5 | 3·6 |
| Bidi | 12 | 4 | 3·0 |
| Cigarette | 3 | 0 | — |
| Other | 3 | 1 | 3·0 |
| Christians | | | |
| Non-smokers | 0 | 1 | — |
| All smokers | 12 | 4 | 3·0 |
| Bidi | 5 | 4 | 1·25 |
| Cigarette | 6 | 0 | — |
| Other | 1 | 0 | — |

Groups I and II, in non-smokers and in smokers identified by religion. Kreyberg's Group I appears to predominate in Hindus, Muslims, and Christians in the overall total number of cases.

The ratio of the number of cases in Kreyberg's Groups I and II compared with the total number of smokers, is

2·6:1. This figure climbs higher if only cigarette smokers are considered (5·7:1). Hindus and Muslims apparently show identical trends. The ratio of cases in Kreyberg's Groups I and II to the number of non-smokers, however, was approximately 1:1 in Hindus, Muslims and the total number of cases.

### DISCUSSION

The quality of our data is satisfactory, as the material is derived from sources of known reliability. It is thus unlikely that the observed low incidence in the Parsis and (to a lesser extent) the other communities could have arisen from any inherent bias in the data.

The age-adjusted incidence in Bombay at 13·6 and 3·3 per $10^5$ males and females respectively, is very low compared with the experience of most other countries (UICC, 1976).

Our male incidence rates are close to those reported from Singapore (Malays, 13·9), New Mexico (Amer. Indians, 12·6), Singapore (Indians, 10·0) and Ibadan (Nigerians, 0·8) reveal even lower rates. On the other hand registries at Liverpool (89·5), Birmingham (77·1), U.S.A. (Detroit (black), 77·1) and Finland (76·5) report 5–7 times higher incidence than in Bombay.

Female incidence in Bombay is comparatively low, being close to that reported from Bulawayo (Africa, 3·1) and Newfoundland (Canada, 3·2). The female population of Malta (1·8) and Warsaw (rural area, 2·5) present even lower rates. In contrast, the Singapore Chinese (17·3) and Birmingham (11·5) experience much higher incidence rates.

Since a high proportion of lung-cancer cases in our data were registered posthumously (19·4% males and 35·1% females), particularly in the older age-groups, the mortality and morbidity rates in Table II are far from being independent measures and any direct comparison between them should thus be avoided.

Our case-control study was restricted to 42·6% interviewed males suffering from lung cancer, as detailed information on tobacco smoking habits was available only from these cases. Failure to interview personally all the patients during the 10-year period cannot bias the results, since the interviewed cases are representative of the total number accepted for study from a known population at risk. Non-availability of an adequate number of beds in our hospitals leads to short periods of admission as in-patients, during which time our medico-social workers could not get to interview all patients prior to their discharge. It is considered unlikely that the patients not personally interviewed would have smoking habits any different from those who were questioned face to face.

The control group was selected by random sampling from the population at risk. Lung cancer cases were matched by age, sex and religion. All the interviews were conducted by trained medico-social workers, and it is thus unlikely that the lung-cancer cases in a hospital setting and the controls in their domestic setting could have produced any bias. As the cancer cases and controls were from the same population at risk they were considered to have been equally exposed to the general environmental hazards, particularly air pollution. The educational and income levels were also similar in the two groups. Smoking was indulged in to a higher degree (81·2%) by the lung-cancer patients than by the controls (21·2%), indicating that cancer cases and controls differ significantly in their smoking habits, although both are from the same population at risk.

The results of the study indicate that tobacco smoked either in the form of a bidi or cigarette is contributory in an equal measure to the development of lung cancer in the Greater Bombay population, in spite of the overall low incidence of the disease. The relative risk of lung cancer in all types of smokers was significantly higher than in non-smokers amongst the Hindu, Muslim and Christian sects, and also when all the communities are taken together. A dose-response relationship in bidi and cigarette smokers was also clearly evident. For all smokers, Kreyberg's Group I type of cancers were preponderant, whereas the ratio of Kreyberg's Group I to II types among non-smokers was found to be equal.

In our data the relative risk of bidi smoking (19·3) was found to be higher than that of cigarette smoking (8·6) in all communities. As the data on bidi and cigarette smoking in the individual communities are inadequate however, we cannot state equivocally which community is basically more exposed to the risk of lung cancer. In fact the relative risk in all types of smokers amongst the Hindus (14·2), Muslims (23·0), Christians (18·0), and all communities taken together (16·8) apparently does not vary to any great extent. Thus Hindus, Muslims and Christians appear to be equally exposed to lung cancer risk from smoking.

Because detailed information is not available on the prevalence of tobacco smoking (both the bidi and cigarette) in the resident population of Bombay by age, sex and religion, we cannot ascertain the specific reasons for the low incidence of lung cancer (in the Parsi and non-Parsi males and females) as compared to Western experience. Whether this situation is due to a difference in the prevalence of the smoking habit, or to any inherent difference in the mode of smoking, or whether it is caused by other environmental factors of genetic susceptibility in the population at risk in the various communities, is not possible to confirm on the basis of the data at our disposal.

Supported in part by Grant NIH-01-006-1 from the National Cancer Institute, Bethesda, Maryland (U.S.A.) and Department of Science and Technology, Government of India, New Delhi, India.

We are grateful to the various hospital administrators, their staff members and medical specialists in private practice who co-operated with us by supplying the required data. Our thanks are due to the Executive Health Officer of the Bombay Municipal Corporation for making available the death records maintained by the Corporation.

We are also grateful to Mr W. I. Lourie, End Results Section, Biometry Branch, National Cancer Institute, Bethesda, Maryland 20014 (U.S.A.) for all the help so readily given to us. We are grateful to the referees for their comments. Messrs M. V. Natekar and T. R. Rajagopalan of the Registry assisted in the data tabulation. Mr R. Ramamurthi typed the manuscript. We thank them all.

### REFERENCES

Census of India (1961) Volume X—Maharashtra Part X (1-B) Greater Bombay Census Tables—PRG. 126(1-B) (Ordinary)/925. Superintendent of census operations, Maharashtra, 1964.

Census of India 1961. Volume X—Maharashtra Part X (1-D). Parsis of Greater Bombay. The Maharashtra census office, Bombay, 1971.

Census of India 1971. Series 11, Part IIA—Maharashtra. General Population Tables. Director of census operations, Maharashtra, 1972.

Jussawalla, D. J., Haenszel, W., Deshpande, V. A. & Natekar, M. V. (1968) Cancer incidence in Greater Bombay: Assessment of the cancer risk by age. *Br. J. Cancer*, **22**, 623.

Jussawalla, D. J. (1975) The persistence of difference in cancer incidence at various anatomical sites 1300 years after immigration. In *Recent Results in Cancer Research*, Vol. 50. Eds E. Grandmann & E. Pederson. Berlin: Springer Verlag. p. 170.

Jussawalla, D. J. & Jain, D. K. (1976) Cancer incidence in Greater Bombay, 1970–72: An epidemiological study. *Indian Cancer Society.*

Jussawalla, D. J. & Jain, D. K. (1977) Breast cancer and religion in Greater Bombay women: An epidemiological study of 2130 women over a 9-year period. *Br. J. Cancer*, **36**, 634.

Kreyberg, L. (1961) Relationship of different histological lung tumour groups to tobacco smoking. *Br. J. Cancer*, **15**, 51.

Mantel, N. & Haenszel, H. (1959) Statistical aspects of the analysis of data from retrospective studies of disease. *J. Natl Cancer Inst.*, **22**, 719.

Notani, P. & Sanghvi, L. D. (1974) A retrospective study of lung cancer in Bombay. *Br. J. Cancer*, **29**, 477.

U.I.C.C. (1976) *Cancer Incidence in Five Continents.* Vol. III. Eds J. Waterhouse, C. Muir, P. Correa & J. Powell. World Health Organisation.